# Self-study tool for integrating health equity into Health in All Policies (HiAP) initiatives

**DOI:** 10.17269/s41997-025-01098-2

**Published:** 2025-09-12

**Authors:** Carol Ragheb, Ketan Shankardass, Laura Lee Noonan

**Affiliations:** 1 Department of Health Sciences, Wilfrid Laurier University, Waterloo, ON Canada; 2 Department of Health and Wellness, Government of Prince Edward Island, Chief Public Health Office, Charlottetown, PE Canada

**Keywords:** Health in All Policies, Public health, Self-study tool, Health, Health equity, Social determinants of health, Intersectoral action, La santé dans toutes les politiques, Santé publique, Autoformation, Santé; Équité en santé, Déterminants sociaux de la santé, Action intersectorielle

## Abstract

**Setting:**

Setting. Health system leaders in Canada recognise that quality improvement alone cannot address health inequities. Intersectoral action, which involves coordination and collaboration across public, private, and third-sector organisations, can improve the distribution of social determinants of health (SDOH) and thereby, health equity. While Health in All Policies (HiAP) promotes this approach, critiques and empirical data highlight implementation gaps over whether health equity is actually being improved. The potential for HiAP initiatives to reduce health inequities can be strengthened by paying greater attention to how these interventions are designed, implemented, and evaluated.

**Intervention:**

We developed and pilot tested a self-study tool that helps organisations learn and reflect on how health equity can be targeted in intersectoral initiatives, including HiAP. This is not the only tool that can be used to consider ways to integrate health equity into intersectoral action; however, it is the first one designed for HiAP initiatives specifically.

**Outcomes:**

The self-study tool asks the user to reflect on a series of health equity concepts to raise awareness about opportunities to better integrate health equity into the design, implementation, and evaluation of intersectoral initiatives.

**Implications:**

The survey and appendix can fill in the gaps of other tools meant to support intersectoral action for health by focusing on ways to strengthen the health equity potential of initiatives. Users can apply the tool prospectively and retrospectively to explicitly target specific criteria to improve how their interventions focus on and potentially address health equity.

**Supplementary Information:**

The online version contains supplementary material available at 10.17269/s41997-025-01098-2.

## Résumé

**Lieu:**

Les responsables des systèmes de santé du Canada reconnaissent que l’amélioration de la qualité ne peut à elle seule aborder les iniquités en santé. C’est par l’action intersectorielle, qui implique une coordination et une collaboration entre les organismes des secteurs public, privé et bénévole, que l’on peut améliorer la distribution des déterminants sociaux de la santé (DSS) et donc l’équité en santé. La santé dans toutes les politiques (SdTP) promulgue une telle approche, mais les critiques et les données empiriques font ressortir des lacunes dans son application et la crainte que l’équité en santé ne soit pas réellement abordée. En faisant plus attention à la conception, à la mise en œuvre et à l’évaluation des initiatives de SdTP, il est possible de renforcer leur potentiel de réduction des iniquités en santé.

**Intervention:**

Nous avons mis au point et testé un outil d’autoformation pour aider les organismes à réfléchir aux moyens de cibler l’équité en santé dans les initiatives intersectorielles comme la SdTP et à en tirer des enseignements. Ce n’est pas le seul outil qui peut servir à envisager des moyens d'intégrer l’équité en santé dans l’action intersectorielle, mais c’est le premier à avoir été conçu spécifiquement pour les initiatives de SdTP.

**Résultats:**

L’outil d’autoformation demande à l’utilisateur d’examiner une série de concepts d’équité en santé pour le sensibiliser aux possibilités de mieux intégrer l’équité en santé dans la conception, la mise en œuvre et l’évaluation d’initiatives de SdTP.

**Implications:**

Le questionnaire et l’annexe peuvent combler les lacunes d’autres outils censés appuyer l’action intersectorielle pour la santé en mettant l’accent sur les moyens de renforcer le potentiel d’équité en santé de diverses initiatives. L’utilisateur peut appliquer l’outil de façon prospective ou rétrospective pour cibler explicitement certains critères afin d’améliorer l’objectif d’une intervention et potentiellement la façon dont elle aborde l’équité en santé.

## Introduction

As health system leaders in Canada have accepted that quality improvement in healthcare alone cannot address persistent and growing health inequities, there has been growing attention to the need to use intersectoral action to improve the distribution of the social determinants of health (SDOH), i.e. the social and economic factors that influence the health and well-being (hereafter simply, health) of an individual (Zygmunt et al., 2019; Boozary & Laupacis, 2020; Tonelli et al., 2020; Martin et al., 2018; Government of Canada, 2024; Kershaw, 2018; Public Health Agency of Canada, 2017). Improving health equity entails fairly distributing resources to improve health in communities experiencing social and/or geographic marginalisation and other barriers to health (Hoyer et al., 2022). As these resources are controlled by various public institutions, private businesses, and third sector organisations, health equity can be strengthened by better coordination and collaboration across these parties— what is often referred to as intersectoral action (Tonelli et al., 2020).

Health in All Policies (HiAP) is a governance approach that relies on intersectoral action to promote health across different sectors by incorporating health considerations in decision-making (Rudolph & Rudolph, 2013). This can put health equity on the agenda for policymakers across sectors, and by extension society-at-large. In Canada, HiAP approaches are increasingly being adopted by governments at the provincial and sub-provincial levels (Province of Quebec, 2023; Van Vliet-Brown et al., 2017).

While HiAP has been promoted as a health equity intervention, some have pointed to an implementation gap as some governments have committed to collaborative actions such as HiAP, but do not carry out initiatives that promote health equity or even measure health equity to begin with (Cairney et al., 2022). This may be for political reasons (Kokkinen et al., 2017a, b), including economic considerations, ideological conflicts, or jurisdictional barriers to upstream action on health equity. Other critiques highlight how HiAP can be used to justify a health or lifestyle drift, where the goal of addressing social inequity in health turns downstream toward addressing intermediary SDOH or health behaviours (Holt & Frohlich, 2022). There is a clear need to strengthen the integration of health equity into the practice of HiAP.

We developed and pilot tested a self-study tool that helps organisations learn and reflect on how health equity can be targeted in intersectoral initiatives, including HiAP. While this is not the only tool that can be used to consider how to integrate health equity into intersectoral action, it is the first one designed for HiAP initiatives specifically. Other relevant tools developed for the advancement of health equity goals include the Health Equity Impact Assessment (HEIA) Tool, n.d., which guides users to consider the impact of their interventions on different vulnerable groups during the planning process to create equitable interventions; and the Health Equity Assessment Tool (HEAT), which supports users during intervention planning through a series of 10 questions based on health equity goals for intervention planning. While these two tools aim to produce equitable interventions prospectively, there remains a need for a tool that considers opportunities for health equity improvement in the context of HiAP initiatives across various stages of design, implementation, and evaluation.

The approach described here addresses this gap by drawing attention to public health concepts and practices relevant to health equity interventions and guiding users to reflect on ways their HiAP initiatives can be improved prospectively or retrospectively. By describing and applying a set of health equity criteria through open-ended and close-ended questions, the tool raises awareness about how health equity can be practically integrated into intersectoral initiatives.

This tool is unique in its application to multiple stages of collaborative action, including more formative work to plan and revise the design of HiAP initiatives, implementation practices, and evaluation activities, as well as design an evaluation of specific intersectoral actions.

This article presents the self-study tool for integrating health equity into HiAP as a fillable survey and an accompanying table containing definitions of and justification for relevant health equity criteria and indicators of criteria implementation. Given this basis, the tool is intended for users with a background in public health. A public health background is useful in fully understanding the terms used in the tool, as well as the implications of health equity in the planning of HiAP interventions.

## Key definitions

### Social determinants of health

The SDOH are non-medical factors that affect individual and population health. The World Health Organization (WHO) defines the social determinants of health as “the conditions in which people are born, grow, live, work and age,” which can affect health behaviours and outcomes (Braveman & Gottlieb, 2014).

Neighbourhood conditions can influence the air and water quality an individual receives and access to nutritional foods, and affects exposure to hazardous substances (Bharmal et al., 2015). An individual’s working conditions also influence their chance of disease; for example, a job that is mainly sedentary can increase the chance of heart disease and obesity (Bharmal et al., 2015). Employment income is also a SDOH, as it determines individual access to material goods and, at times, the quality of healthcare. Education can influence health by shaping an individual’s health knowledge, literacy, and perceived personal control. Many of the SDOH can be influenced by government policy and decision-making, and by decisions of extra-governmental actors, making it important for policymakers and government leaders to consider the effects of different sectors on health. In this way, HiAP is a health equity intervention that addresses the SDOH.

### Health equity

Health equity can be defined as a state where every individual in a society can achieve their full potential for health (Liburd et al., 2020). Health inequities can be avoided and are shaped by the political, social, and economic resources where individuals live, work, and grow (World Health Organisation, 2022). In order to improve health equity, the WHO recommends improving daily living conditions, addressing the inequitable distribution of power and resources, and measuring health inequities’ impact on policymaking (World Health Organisation, 2022). Health disparities (or inequalities) can be used as a measure of progress toward health equity, as they measure the differences that are caused by economic, social, or environmental disadvantages (Jackson & Huston, 2016).

The state of health equity in a society can be understood as a function of the distribution of SDOH. This distribution is influenced by structural determinants, including policies and governance arrangements that reflect political, legal, and economic hierarchies (Baciu et al., 2017). Structural determinants of health can simultaneously operate at municipal, provincial, or federal levels, while specific SDOH are often controlled in large part by specific levels of governments. For example, in Canada, education system policy and curriculum is mainly a provincial/territorial responsibility, while primary and secondary school systems are operated at more local jurisdictions (e.g. by school boards). The federal government plays less of a role in the education system (mainly funding), except in the case of its direct responsibility to fund and oversee education for certain Indigenous communities (e.g. children on First Nations reserves). Therefore, intersectoral action is needed at all levels of government to strengthen health equity.

## Tool development

To develop the self-study tool, a list of health equity criteria (Table 1) was first developed based on a review of existing literature about health equity interventions. The list of health equity criteria was divided into four categories: goals of the collaboration, identification of inequities, intervention planning, and intervention evaluation. These four categories reflect key opportunities to embed an intentionality to improve health equity in the use of HiAP. Alongside the health equity criteria, implementation indicators and justification of each criterion were also developed based on existing literature, as shown in Appendix A. Health equity criteria informed the design of open-ended and/or closed-ended questions to prompt users to reflect on the health equity potential of their current approach and how to strengthen it through changes to the design, implementation, and/or evaluation of HiAP.

**Table 1:** Health equity criteria across four categories of HiAP activities

Collaborative action goals	- Broadly inclusive definition of health and well-being - Language reflecting health equity concepts
Identifying health inequalities	- Evidence used to identify health inequalities - Analysis to identify demographic patterns and social determinants - Reflection on trends over time
Intervention planning	- Equity rationale for interventions - Aim to reduce health inequalities - Involvement of knowledge holders in planning - Distribution of power across knowledge holders - Implementation of upstream interventions - Multidimensional intervention approaches
Evaluation of interventions	- Ongoing evaluation over time - Modification of actions based on evaluation results

A pilot study of the health equity tool took place over the course of four months with public health practitioners involved in HiAP, as well as academic researchers focused on HiAP. Practitioner feedback was essential given that they are the target audience, while academic researchers can have expertise in how to design and evaluate health equity interventions. Pilot testers were sent the health equity tool (i.e. survey and appendix) and asked to review it during a two-week period before taking part in a debrief meeting to discuss feedback. Participant feedback directly informed revisions to the tool.

The recruitment of pilot testers was done through a web search of relevant academic researchers and public health practitioners; through an email to the members of the Canadian Network for Health in All Policies; and through snowball sampling. For the first method, a web search of Canadian researchers and practitioners was conducted, and an email was sent to 13 individuals explaining the study and how they can become participants. For the second method, the same email was sent to 14 members of the Canadian Network for Health in All Policies. Both emails contained a message asking recipients to forward the invitation to anyone they may know who fits the inclusion criteria.

After getting responses from participants, there were five meetings held with seven participants who acted as pilot testers and reviewers of the tool. This included public health practitioners (3), public health policymakers (2), a public health nurse (1), and a researcher (1), all with experience and expertise related to intersectoral action for health equity. The pilot testing took place from August 2023 to October 2023, after which the feedback was reviewed and incorporated into the final tool.

## Application of tool

### Recommended users

The target users for the self-study tool are public health actors involved in collaborative action, since they will have expertise about the concepts underlying the criteria in this tool and how they apply to health equity interventions like HiAP. Having some experience with intersectoral collaboration will also increase the familiarity of relevant concepts to users, as will experience with intervention design and evaluation.

### How to use the tool

The self-study tool for integrating health equity into HiAP action is a fillable survey accompanied by an appendix. Although interventions are often developed and refined through feedback (e.g. from evaluation) in a cyclic manner and with overlap across stages of work, the survey is organised in a more simplified chronological order to make it more feasible to use, thereby increasing uptake by the intended audience. The survey starts with questions about the definition of goals for the HiAP initiative and the identification of inequities, and continues through topics related to the planning and evaluation of interventions that arise from HiAP.

Typically, survey questions ask a user to reflect and report on how their HiAP initiative has addressed a particular criterion to date, and there are then follow-up questions that give the user an opportunity to reflect on how health equity could be better integrated moving forward. Users can complete the tool on their own but will also find value in discussing certain questions with other collaborators who could know more about certain aspects of the HiAP initiative. After completing the survey, users are encouraged to reflect on their answers and share any takeaways with their collaborators to create conversations about improving the health equity potential of their approach.

While completing the tool, users should also refer to the appendix, which contains a table of information about the health equity criteria that underlie specific questions in the survey. This provides a valuable summary of the ways that HiAP initiatives can work toward health equity. The information in this table supports the user with a deeper understanding of the criteria being assessed, and related indicators of strong approaches to health equity in HiAP practice. The table also includes a justification for why these indicators matter, which can inform the management of HiAP practice, while also informing discussions among collaborators about how to make changes to a HiAP initiative to improve the ways it is addressing health equity.

### When to use the tool

Health in All Policies initiatives can be understood as developing over time with growing maturity (Storm et al., 2014). They may start as responsive forms of collaboration where the importance of intersectoral action to address health equity is recognised and initial actions are being planned and implemented, before becoming more proactive or embedded governance projects, where deeper investments in intersectoral action are being made and processes for collaboration are being refined and institutionalised (Storm et al., 2014). Since this tool can be used proactively to integrate equity considerations in a relatively new HiAP initiative, or retrospectively to fine-tune an existing initiative, it can be applied by users involved in HiAP across a range of maturity levels.

The self-study tool can be used at the beginning of intervention planning, such as when organisations are starting to design interventions or are seeking ways to strengthen their interventions. It can also be used as a reflective exercise, at any point of intervention planning, to evaluate progress in implementing the health equity criteria. For example, it can be used after interventions have been implemented and need evaluation.

## Discussion

Although the concept of HiAP includes a focus on addressing health inequities in theory, evidence from places where a HiAP approach has been applied suggests that impacts on health equity are less reliable (World Health Organisation*,* 2023; Hall & Jacobson, 2018; van Eyk et al., 2017). Although some evidence suggests that political decisions may drive some of these failures, there has also been a lack of guidance for how to comprehensively consider ways to embed health equity when designing, implementing, and evaluating HiAP initiatives (Kokkinen et al., 2017b). Moreover, initiatives that can be classified as HiAP due to the use of sustained multisectoral collaboration on determinants of health may not have been intentionally designed using HiAP principles and therefore may not have initially had health equity in mind as a key objective.

This self-study tool will allow organisations involved in HiAP-like initiatives to more intentionally integrate health equity principles, both prospectively and retrospectively. By engaging in the tool, users will be able to learn about relevant criteria and indicators when defining goals of collaborative action, identifying the inequities, and planning for interventions and evaluation. Looking back, the tool will help users evaluate their approach to health equity and reflect on ways to improve.

In general, the tool does not provide specific ways of improving the design of HiAP initiatives. For example, we ask users to reflect on the goals of their initiatives and suggest that they be “modified to include a more holistic definition of health and well-being”; however, it does not prescribe whether that means using the WHO definition, or one that is created locally to garner buy-in from the specific array of sectors participating in the initiative. This is intentional on our part since every HiAP initiative serves a unique context, including historical, cultural, economic, and other social realities, which require careful consideration about how to make HiAP work best over time. This is reflected in the new WHO “four pillars” approach to designing HiAP initiatives, which highlights functional elements of importance (i.e. governance and accountability; leadership at all levels; methods of work and ways of working; and resources, financing, and capabilities) (World Health Organisation, 2023), but does not define what they ought to look like in a given setting. In other words, the users of this tool and their collaborators are the best people to determine the specific ways to integrate health equity into their HiAP initiative. In addition to looking for peer-reviewed articles that may share ideas for specific approaches, users can look to repositories of HiAP and public health resources, such as those hosted by PAHO/WHO, the Global and Canadian Networks for HiAP, and the National Collaborating Centres in Canada (National Collaborating Centre for Methods and Tools, 2013; Pan American Health Organisation, n.d.; National Collaborating Centre for Healthy Public Policy,  2024); Action: SDG – All for Equity – Convening SDH Actors to Reach the SDGs, 2019; Canadian Network for Health in All Policies, n.d.).

In summary, the strengths of this self-study tool are that it offers a quick and relatively low-burden way to comprehensively identify ways that a HiAP initiative can be designed or modified to strengthen the health equity potential. The tool is also flexible in that it can be used prospectively, when designing a new HiAP initiative, or retrospectively to reflect and improve an existing initiative. Finally, the tool is designed around a theoretical framework of health equity criteria and indicators that have been individually developed and utilised elsewhere.

Important limitations include that a person with a public health background may be required to lead the use of the tool given the concepts and language used in the underlying criteria and indicators, and therefore the tool itself. This may be inconvenient in settings where public health personnel are limited or non-existent. Another challenge is that the usage of the tool to strengthen the health equity potential of a HiAP initiatives may not be politically or economically feasible. Holt and Frohlich (2022) highlight the need for public health actors to advocate more strongly for changes to the “core missions and services” (p. 281) of non-health sectors, and it is unclear if the tool can achieve that objective even as it raises the importance of designing interventions that work through those sectors. In turn, some of the plans for altering the design of a HiAP initiative that emerge from use of the tool may require frank discussions about the importance and logic behind such changes and strategic advocacy (e.g. win–win approaches) to gain buy-in.

## Implications for policy and practice

What are the innovations in this policy or program? Explain why this policy or program is considered an innovation. Example responses: applying/adapting an accepted practice or model to a novel problem; development of a new practice or model with preliminary evidence of success; challenging the prevailing scope and practice of public health with corresponding rationale; etc.

- This self-study tool supports innovative public health practice in the use of Health in All Policies (HiAP). While HiAP is promoted as a health equity intervention, there is little guidance on how to intentionally integrate health equity in the design, implementation, and evaluation of HiAP. The tool addresses this gap by bringing together existing and novel health equity concepts and formulating them into a series of reflective questions for public health actors. Finally, some HiAP advice implies that HiAP initiatives are static in design; the tool acknowledges the developmental nature of HiAP by being applicable to initiatives across a range of stages.

What are the burning research questions for this innovation? i.e., What would be needed to scale up the innovation, and/or do rigorous evaluation research to corroborate preliminary findings?

- Do public health actors find the tool useful in a wide range of HiAP contexts?
- Since the tool is not prescriptive, how can public health actors use the information generated by the tool to improve the health equity potential of their interventions in a specific way?
- Does use of the tool lead to design changes that improve health equity in the population and in settings where HiAP is practiced?

## Supplementary Information

Below is the link to the electronic supplementary material.

ESM 1 (DOCX 85.9 KB)

## Data Availability

Not applicable.

## References

[CR1] Action: SDG – All for Equity – Convening SDH Actors to Reach the SDGs. (2019). Retrieved from August 13, 2025 from Ku.edu. https://actionsdg.ctb.ku.edu/

[CR2] Baciu, A., Negussie, Y., Geller, A., & Weinstein, J. N. (2017, January 11). *The root causes of health inequity*. Nih.gov; National Academies Press (US). https://www.ncbi.nlm.nih.gov/books/NBK425845/

[CR3] Bharmal, N., Derose, K., Felician, M., Weden, M., & Health, R. (2015). *Understanding the upstream social determinants of health*. https://www.rand.org/content/dam/rand/pubs/working_papers/WR1000/WR1096/RAND_WR1096.pdf?utm_source=miragenews&utm_medium=miragenews&utm_campaign=news

[CR4] Boozary, A., & Laupacis, A. (2020). The mirage of universality: Canada’s failure to act on social policy and health care. *Canadian Medical Association Journal,**192*(5), E105–E106. 10.1503/cmaj.200085 32015078 10.1503/cmaj.200085 PMC7004220

[CR5] Braveman, P. (2006). Health disparities and health equity: Concepts and measurement. *Annual Review of Public Health,**27*(1), 167–194. 10.1146/annurev.publhealth.27.021405.102103 16533114 10.1146/annurev.publhealth.27.021405.102103

[CR6] Braveman, P., & Gottlieb, L. (2014). The social determinants of health: It’s time to consider the causes of the causes. *Public Health Reports,**129*(Suppl 2), 19–31. 24385661 10.1177/00333549141291S206 PMC3863696

[CR7] Cairney, Paul, et al. (2022). Why is health improvement policy so difficult to secure? *Open Research Europe,**2*, 76. 10.12688/openreseurope.14841.2 37645286 10.12688/openreseurope.14841.2 PMC10445925

[CR8] Canada. (2024). *A healthy, productive Canada : A determinant of health approach / The Honourable Wilbert Joseph Keon, Chair; The Honourable Lucie Pépin, Deputy Chair. : YC17–402/3–01E-PDF - Government of Canada Publications - Canada.ca*. Publications.gc.ca. Retrieved August 12, 2025, from https://publications.gc.ca/site/eng/359901/publication.html

[CR9] Canadian Network for Health in All Policies (2025, August 13). (n.d.)*National Collaborating Centre for Healthy Public Policy.* Retrieved , from https://ccnpps-ncchpp.ca/canadian-network-for-health-in-all-policies-cnhiap/

[CR10] Hall, R. L., & Jacobson, P. D. (2018). Examining whether the health-in-all-policies approach promotes health equity. *Health Affairs,**37*(3), 364–370. 10.1377/hlthaff.2017.1292 29505382 10.1377/hlthaff.2017.1292

[CR11] Health Equity Assessment Toolkit. (n.d.). Www.who.int https://www.who.int/data/inequalitymonitor/assessment_toolkit

[CR12] Health Equity Impact Assessment (HEIA). (n.d.). CAMH. Retrieved August 12, 2025, from https://www.camh.ca/en/professionals/professionals--projects/heia

[CR13] Health in All Policies. (2022, December 13). National Collaborating Centre for Healthy Public Policy. Retrieved August 13, 2025, from https://ccnpps-ncchpp.ca/health-in-all-policies/

[CR14] Holt, D. H., & Frohlich, K. L. (2022). Moving beyond health in all policies: Exploring how policy could front and centre the reduction of social inequities in health. *Integrating Science and Politics for Public Health*, 267–291. 10.1007/978-3-030-98985-9_12

[CR15] Hoyer, D., et al. (2022) How do we define and measure health equity? The state of current practice and tools to advance health equity. *Journal of Public Health Management and Practice*, *28*(5), 570–577. journals.lww.com/jphmp/Fulltext/2022/09000/How_Do_We_Define_and_Measure_Health_EquityThe.20.aspx, 10.1097/PHH.0000000000001603 10.1097/PHH.0000000000001603 PMC9311469 35867507

[CR16] Jackson, B., & Huston, P. (2016). Advancing health equity to improve health: The time is now. *Health Promotion and Chronic Disease Prevention in Canada : Research, Policy and Practice,**36*(2), 17–20. 26878490 10.24095/hpcdp.36.2.01 PMC4910425

[CR17] Kershaw, P. (2018). The need for health in all policies in Canada. *Canadian Medical Association Journal,**190*(3), E64–E65. 10.1503/cmaj.171530 29358199 10.1503/cmaj.171530 PMC5780264

[CR18] Kokkinen, L., Muntaner, C., O’Campo, P., Freiler, A., Oneka, G., & Shankardass, K. (2017a). Implementation of Health 2015 public health program in Finland: A welfare state in transition. *Health Promotion International,**34*(2), 258–268. 10.1093/heapro/dax081 10.1093/heapro/dax081 29149295

[CR19] Kokkinen, L., Shankardass, K., O’Campo, P., & Muntaner, C. (2017b). Taking health into account in all policies: Raising and keeping health equity high on the political agenda. *Journal of Epidemiology and Community Health,**71*(8), 745–746. 10.1136/jech-2016-207736 28416570 10.1136/jech-2016-207736

[CR20] Liburd, L. C., Hall, J. E., Mpofu, J. J., Marshall Williams, S., Bouye, K., & Penman-Aguilar, A. (2020). Addressing health equity in public health practice: Frameworks, promising strategies, and measurement considerations. *Annual Review of Public Health*. 10.1146/annurev-publhealth-040119-094119 31900101 10.1146/annurev-publhealth-040119-094119

[CR21] Martin, D., Miller, A. P., Quesnel-Vallée, A., Caron, N. R., Vissandjée, B., & Marchildon, G. P. (2018). Canada’s universal health-care system: Achieving its potential. *The Lancet,**391*(10131), 1718–1735. 10.1016/S0140-6736(18)30181-8 PMC7138369 29483027

[CR22] National Collaborating Centre for Healthy Public Policy (2024). Stronger together! Five policy briefs about strengthening implementation of Health in All Policies (HiAP) at the local level in Ontario and Qu?bec. Retrieved August 13, 2025, from https://ccnpps-ncchpp.ca/stronger-together-five-policy-briefs-about-strengthening-implementation-of-health-in-all-policies-hiap-at-the-local-level-in-ontario-and-quebec/

[CR23] National Collaborating Centre for Methods and Tools. (2013). Nccmt.ca. Retrieved August 13, 2025, from https://www.nccmt.ca/

[CR24] Pan American Health Organization. (n.d.). Health in all policies - PAHO/WHO. Retrieved August 13, 2025, from https://www.paho.org/en/topics/intersectoral-action-health-all-policies

[CR25] Province of Quebec (2023, August 2) *- Global Network for Health in All Policies*. Global Network for Health in All Policies. Retrieved August 13, 2025, from https://actionsdg.ctb.ku.edu/network-members/province-of-quebec/

[CR26] Public Health Agency of Canada. (2017). The Chief Public Health Officer’s Report on the State of Public Health in Canada 2017 –*Designing healthy living* - Canada.ca. Retrieved August 13, 2025, https://www.canada.ca/en/public-health/services/publications/chief-public-health-officer-reports-state-public-health-canada/2017-designing-healthy-living.html

[CR27] Rudolph, L. I., & Rudolph, S. H. (2013). Health in all policies: Improving health through intersectoral collaboration. *Studies in Indian Politics*, vol. 1, no. 1, June 2013, 10.1177/2321023013482782.

[CR28] Storm, I., Harting, J., Stronks, K., & Schuit, A. J. (2014). Measuring stages of health in all policies on a local level: The applicability of a maturity model. *Health Policy,**114*(2–3), 183–191. 10.1016/j.healthpol.2013.05.006 23764153 10.1016/j.healthpol.2013.05.006

[CR29] Tonelli, M., et al. (2020). Canada needs a “Health in All Policies” action plan now. *Canadian Medical Association Journal,**192*(3), E61–E67. 10.1503/cmaj.190517 31959656 10.1503/cmaj.190517 PMC6970602

[CR30] van Eyk, H., Harris, E., Baum, F., Delany-Crowe, T., Lawless, A., & MacDougall, C. (2017). Health in all policies in South Australia—did it promote and enact an equity perspective? *International Journal of Environmental Research and Public Health,**14*(11), Article 1288. 10.3390/ijerph14111288 29068400 10.3390/ijerph14111288 PMC5707927

[CR31] Van Vliet-Brown, C. E., Shahram, S., & Oelke, N. D. (2017). Health in all policies utilization by municipal governments: Scoping review. *Health Promotion International,**33*(4), 713–722. 10.1093/heapro/dax008 10.1093/heapro/dax008 28334905

[CR32] World Health Organization (WHO). (2023). *Working together for equity and healthier populations Sustainable multisectoral collaboration based on health in all policies approaches.* Retrieved August 13, 2025, from https://iris.who.int/bitstream/handle/10665/372714/9789240067530-eng.pdf?sequence=1

[CR33] World Health Organisation. (2022). *Health Equity -- Global*. Retrieved August 12, 2025, from www.who.int. https://www.who.int/health-topics/health-equity#tab=tab_1

[CR34] Zygmunt, A., Kendall, C. E., James, P., Lima, I., Tuna, M., & Tanuseputro, P. (2019). Avoidable mortality rates decrease but inequity gaps widen for marginalized neighborhoods: A population-based analysis in Ontario, Canada from 1993 to 2014. *Journal of Community Health,**45*(3), 579–597. 10.1007/s10900-019-00778-8 10.1007/s10900-019-00778-8 31722048

