# Supplementary material for: Self-study tool for integrating health equity into Health in All Policies (HiAP) initiatives
Source: Can J Public Health. 2025 Sep 12;117(2):384–90. doi: 10.17269/s41997-025-01098-2 (PMC13076818; doi:10.17269/s41997-025-01098-2)
Supplement: Supplementary file 1 — (DOCX 85.9 KB) [file 41997_2025_1098_MOESM1_ESM.docx]

Self-Study Tool for Integrating Health Equity into Health in All Policies (HiAP) Initiatives, Canadian Journal of Public Health

Self-Study Tool for Embedding Health Equity into HiAP

Health in All Policies (HiAP) is an approach to collaborative governance that uses sustained intersectoral action to address social determinants of health and improve health equity. Each HiAP initiative is designed in a unique way and implemented in a unique setting. This self-study tool aims to give public health actors engaged in HiAP initiatives an understanding of how health equity has been addressed in their activities to date, and how health equity can be better integrated into activities moving forward. The tool can also inform the design of a new HiAP initiative with health equity in mind.

There are four sections contained in the survey portion of the tool, as well as the sections of the appendix The first section considers the extent to which health equity is indicated in the goals of the initiative. The second section examines the ways that health inequities are identified for remediation by the initiative over time. The third section examines the ways that population interventions are designed and implemented. The fourth section examines the ways that these initiatives are evaluated.

Across sections, this tool includes a series of close-ended and open-ended questions.

For simplicity, the questions often refer to your HiAP initiative simply as a “collaboration." One person on your team should take responsibility for completing the tool, and because of the concepts being discussed, we recommend that this person have a background in public health. It will be helpful to consult with others involved in your initiative to respond to some questions. We expect the tool will take around 90 minutes to complete.

After using this tool, users may seek additional resources to continue strengthening the health equity potential of their initiative based on their needs. If you have any additional questions, you can contact Dr. Ketan Shankardass [(kshankardass@wlu.ca).](mailto:(kshankardass@wlu.ca)

# Section 1: Goals of the Collaboration

Organizations using a HiAP approach can define their goals using *a broad, holistic definition of health and well-being* so that interventions do not end up narrowly focused on addressing disease outcomes. Further, the *intention to improve equity through collaborative action* can be made explicit in goals to clearly communicate to partners this key focus of the collaboration.

The focus on equity in the collaboration can be reinforced by integrating other equity-related goals that may be pursued by parties in your jurisdiction, such as IDEA (inclusion, diversity, equity and accessibility) or sustainability.

1. What are the stated goals of the collaboration? Are there any other equity-related goals being pursued in your jurisdiction that could be integrated into the goals of the collaboration?

2a. Could the goals of the collaboration be improved in some way to include a more holistic definition of health and well-being? Health is defined by the WHO as “a state of complete physical, mental and social well-being and not merely the absence of disease or infirmity.”

- -
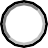
Yes
  -
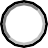
Maybe
  -
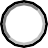
No

If **Yes/Maybe** selected:

2b. How could the goals be modified to include a more holistic definition of health and well-being?

3a. Could the goals of the collaboration be improved to include language that indicates the improvement of equity in the population more explicitly? For example, this may include using language such as improving population health or local conditions for “everyone”, or identifying specific population groups, such as children, the elderly or women who are refugees, who are being targeted for improvement.

- -
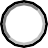
Yes
  -
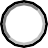
Maybe
  -
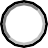
No

If **Yes/Maybe** selected:

3b. How could the goals be modified to include language that indicates the improvement of equity in the population?

# Section 2: Identification of Inequities

The World Health Organization defines equity as, “the absence of avoidable or remediable differences among groups of people, whether those groups are defined socially, economically, demographically, or geographically”. Therefore, measuring differences in health outcomes between groups of people is an important step for identifying equity-deserving target groups and considering relevant interventions.

The measurement of health differences can be relatively simple (i.e., comparison between two groups; e.g., rates of heart disease are higher in men compared to women) or more stratified or intersectional (i.e., subgroups are explored to identify specific vulnerable groups; e.g., racialized men of lower SES have higher rates of heart disease compared to non-racialized men and those of higher SES).

To understand how specific inequities are changing over time, possibly in response to interventions, it can be useful to measure health differences at multiple time points. To better understand risk factors underlying inequities and start thinking about interventions, it can also be useful to characterize identified target groups in terms of social determinants of health (e.g., housing, employment, race, gender and social exclusion) and other risk factors.

In summary, when identifying inequities, evidence can be used to:

- identify health differences based on one or more characteristics (e.g., race/ethnicity, sex, gender identity, age, socioeconomic status, geographic location).
- identify relevant risk factors to target for improvement in interventions.
- track health differences over time.

Collaborations can rely on several existing tools, such as [health equity impact assessment](https://www.camh.ca/en/professionals/professionals--projects/heia), which can be used to identify health inequities and relevant risk factors

4a. Does the collaboration use evidence about differences in health and well-being in the population to identify target groups in the community that require intervention?

- -
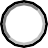
Yes
  -
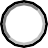
Maybe
  -
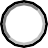
No

If **No** selected:

4b. What strategies could be adopted to aid in the identification of target groups?

If **Yes/Maybe** selected:

4b. Briefly summarize how evidence about differences in health and well-being has been used to identify target groups for equity improvement? Please include any detail about the type of stratification used (i.e., social, economic, demographic, geographic) and about how often evidence about differences are measured over time (if at all) to identify target groups.

4c. To what extent were analyses of risk factors such as the social determinants of health used to better understand the underlying issues affecting target groups? Please include any details about the risk factors examined.

4d. In what ways could the identification of target groups and understanding of inequities be improved, for example by examining other population stratifiers or risk factors?

4e. Could the collaboration make better use of tools that may help identify target groups and improve understanding of how to improve equity, such as the [health equity impact](https://www.camh.ca/en/professionals/professionals--projects/heia) assessment tool?

# Section 3: Intervention Planning

There are a variety of ways that intersectoral committees may engage in planning what to collectively target to work upstream on improving population health and well-being; however, it is important to include a focus on improving equity. Ideally, the process will include consideration of evidence about the specific inequities being experienced by various groups and communities and learning from examples of how prevention has helped address similar problems experienced elsewhere.

While planning health equity interventions, it can be useful to develop theories of change that consider the specific contributions of various partners over time, to implement the intervention and consider equity goals in the short and long-term. [Logic models](http://www.publichealthontario.ca/-/media/documents/focus-on-logic-model.pdf) can be used as a tool in planning intervention by allowing users to articulate the mechanisms that would need to be triggered for an intervention to work, and what success will look like over time. This can provide a more realistic understanding of the intervention, including the processes and resources required for the intervention to work, and expected outcomes to partners involved. It can also be useful to predict possible barriers to planning, such as access to resources, to achieve successful outcomes and work to resolve barriers in implementation of interventions.

5a. What are the ways that intervention planning has been conducted?

5b. How could the process of planning equity-focused interventions be improved in your collaboration? Are there ways to increase the use of evidence, or more clearly articulate how and why interventions are meant to work?

6a. Are improvements in equity explicitly targeted when planning interventions in the collaboration?

-
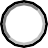
Yes
-
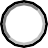
Maybe
-
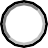
No

6b. How could the targeting of health equity improvement be strengthened? What are some of the barriers to having a stronger focus on equity?

When planning interventions, consultation with a variety of knowledge holders can be useful, for example to more clearly define the problem, better plan the intervention, and understand how to evaluate the intervention. This may include consultation with people with lived or living experience, community leaders, academics, and others who may have expertise or valuable opinions.

It can also be useful to consider who has power in decision making, and whether the input of knowledge holders is integrated in the planning of interventions. Gaining the opinion and lived experiences from members of target populations can be helpful in predicting barriers to interventions and understanding how to best address inequity.

7a. During intervention planning, what types of knowledge holders were engaged? If a variety of parties have been engaged when planning different interventions, you can describe that.

7b. Are there any knowledge holders not yet included in intervention planning who could be engaged to improve your understanding of how to improve health equity?

8a. When making decisions in the collaboration, how was the control of decision making divided between the knowledge holders? For example, did people with lived and living experiences have some control over the decisions, or were they simply consulted (e.g., was their input incorporated into decisions?)

8b. How can the control of decision making be shared more equally across knowledge holders?

When developing equity-focused interventions, it can be most effective to use an *upstream* approach that targets structural determinants such as social status, income, racism, and exclusion to prevent health inequities before they arise. These factors may be addressed through policies and programs implemented by the government or other institutions. For example, an upstream approach to addressing inequities in nutrition-related illnesses could be to include a basic income program that provides all families with adequate financial security to purchase more nutritious ingredients and prevent the onset of disease.

Interventions may also use a *midstream* approach to reduce the risk of disease outcomes manifesting and inequities arising. For example, a midstream approach to addressing inequities in nutrition-related illnesses could include a training program to teach lower income families how to prepare nutritious meals with affordable ingredients.

Finally, *downstream* approaches address the health care needs of a target group after health inequities have already manifest, including the accessibility and quality of care. For example, a downstream approach to addressing nutrition-related illnesses could include improving access to healthcare for lower income people living with these illnesses to improve their prognosis.

A *multidimensional* approach that encompasses multiple interventions that include different entry points (upstream, midstream, downstream) can more effectively address the needs of a target group.

9a. When you think about the interventions implemented by the collaboration to date, did they include upstream approaches?

-
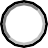
Yes
-
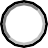
Maybe
-
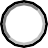
No

If **Maybe/No** is selected:

9b. In what ways could upstream approaches be incorporated in future interventions?

10a. When you think about the interventions implemented by the collaboration to date, did they include midstream approaches?

-
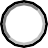
Yes
-
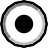
Maybe
-
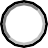
No

If **Maybe/No** is selected:

10b. In what ways could midstream approaches be incorporated in future interventions?

11a. When you think about the interventions implemented by the collaboration to date, did they include downstream approaches?

-
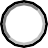
Yes


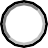


o

Maybe
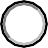
 No

If **Maybe/No** is selected:

11b. In what ways could downstream approaches be incorporated in future interventions?

# Section 4: Evaluation of Interventions

It is important to evaluate interventions to understand the effects they are having on inequalities (outcome evaluation). It can also be useful to measure implementation processes to understand if the intervention is being implemented as designed (process evaluation). These and other approaches to evaluation can provide insight about the strengths and weaknesses of an equity intervention and inform improvements to current and future interventions.

When evaluating equity interventions, it can be helpful to track processes and inequalities to understand if equity is being strengthened over time, and if not, why not. If a logic model exists or can be developed about interventions, they can be relied on to carefully consider what to measure over what timeline.

12a. Were interventions that emerged from the collaboration evaluated over time?

-
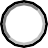
Yes
-
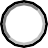
Maybe
-
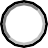
No

If **Yes/Maybe** is selected:

12b. Please briefly summarize the range of evaluations that have been used, including any specific indicators and metrics used:

13. Are there any other ways that evaluation processes could be improved?

14a. Is evidence from evaluation used to improve existing interventions or introduce new interventions over time?

-
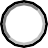
Yes
-
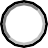
Maybe
-
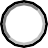
No

14b. How could the improvement of interventions based on evidence from evaluation take place?

# Appendix: Health Equity Criteria for Self-Study Tool for Integrating Health Equity into HiAP

The following table includes information to describe how the self-study tool evaluates the integration of health equity in Health in All Policies (HiAP).

- The first column identifies relevant questions from the tool.
- The second column describes the health equity criteria that our conceptual framework indicates ought to be integrated into HiAP initiatives.
- The third column describes indications of whether or not these criteria are present in a specific HiAP initiative, and can therefore serve to guide the user on how to strengthen the health equity potential of a HiAP initiative.
- The fourth column justifies the relevance of the criteria.

This table can be used during completion of the self-study tool to clarify specific questions, as well as after completing the self-study tool to reflect on the answers generated while completing it.

| **Questions from Tool** | **Health Equity Intervention Criteria in Collaboration** | **Indications of Criteria Implementation** | **Justification of Criteria** |
| --- | --- | --- | --- |
|  | ***Section 1. Goals of the collaboration*** | | |
| 1, 2a, 2b | Use of a broadly inclusive definition of health and well- being in goals of collaborative action. | Health is described using a concept that implicitly includes: physical, mental, social, environmental, and spiritual well- being (e.g., quality of life). | Using a more holistic approach to health can result in collaborative action that more comprehensively addresses  the drivers of health inequity. |
| 3a,3b | Use of language that reflects health equity concepts in goals of collaborative action. | Goals indicate:   - a desire to improve health for “everyone” and/or reduce gaps between groups and/or address a social gradient. - a need to address unfair or unjust differences. | The goal of equity should be clearly communicated to partners so that collaborative action can focus on equity improvement and not only population health improvement. |
|  | ***Section 2. Identification of inequities*** | | |

1

| 4a,4b,4e | Use of evidence to identify health inequalities, including gaps and gradients along one or more characteristics (e.g., cardiovascular rates among lower versus high income women). | Evidence used to identify health differences based on one or more characteristics (e.g., race/ethnicity, sex, gender identity, age, socioeconomic status, geographic location).  Tools like HEIA may be used to identify population groups of interest. | Using evidence to identify population groups in need is a rigorous way to determine how to target collaborative action. |
| --- | --- | --- | --- |
| 4a,4b, 4c, 4d | Additional analysis of health inequalities to identify patterns of demographics and risk factors, including social determinants of health. | A range of evidence (e.g., qualitative, quantitative) used to identify specific subgroups with the highest levels of health inequality and relevant risk factors. | Subgroup analysis of health inequalities can enable a clearer understanding of which population groups to target in collaborative action, and possible underlying causes of the inequity. |
| 4a,4b | Repeated measurement of inequalities over time. | Evidence used to identify and track specific inequalities at multiple time points. | Tracking inequalities over time as collaborative action is undertaken enables evaluation and revision of intervention approaches. |
|  | ***Section 3. Intervention***  ***planning*** |  |  |
| 5a | Justification of interventions. | Interventions designed based on evidence from successful models relevant to identified inequalities.  Theory of change (including logic models) applied in designing interventions. | Using examples of success for health equity interventions can make them more credible to partners and provide guidance on how to adapt examples for local use.  Using theories of change can clarify the reasoning behind how an intervention will work through collaborative action, and  enable appropriate evaluation. |
| 5b, 6a, 6b | Interventions explicitly  target reduction in health inequalities. | Intervention is designed to reduce health inequalities, not just general population health | Interventions that do not include targeting of population groups with the |

2

|  |  | improvement. | most need can end up widening health inequalities, e.g., if less marginalized communities take advantage of resources  being introduced. |
| --- | --- | --- | --- |
| 7a, 7b | Consultation of knowledge holders during intervention planning. | Intervention planning process includes input from relevant knowledge holders such as: people with lived and living experience, service providers, academic experts. | Knowledge about the nature of the problems causing inequity and which solutions may best address the underlying causes may be held by a range of people. Exclusion of relevant perspectives can result in a poorly designed or otherwise ineffective  intervention. |
| 8a, 8b | People with lived and living experience given power during intervention planning. | Evidence that members of target population groups had some control over the intervention planning process. | Communities affected by health inequity have valuable knowledge about the problems causing inequity and which solutions may best address the underlying causes; and their voices can be discounted by more powerful actors in the  intervention planning process. |
| 9a, 9b | Implementation of upstream interventions. | Inequity is not being addressed solely through interventions that target amelioration of health inequalities after they manifest. Interventions also includes focus on preventing inequity before or during the onset of causal processes. | Addressing the root causes of inequity (e.g., by addressing social factors, like housing, education, employment) can be the fairest and most effective since they can reduce the unequal distribution of harm in the first place. |
| 10a, 10b,  11a, 11b | Multidimensional approach in interventions. | Evidence that collaborative action includes multiple activities that target different causal | Most health inequities result from a confluence of causal processes, so a more |

3

|  |  | processes of inequity. | multidimensional approach can be more useful in preventing inequity. |
| --- | --- | --- | --- |
|  | ***Section 4: Evaluation of Interventions*** |  |  |
| 12a, 12b, 13 | Evaluation of interventions over time. | Evidence of potentially multiple evaluation processes that examine process and outcome indicators of equity interventions over time.  Theory of change (including logic models) applied in evaluating interventions. | Evaluation of interventions can provide insight about strengths and weakness of interventions, and provide guidance about how to revise existing interventions or introduce new interventions to more effectively address inequities.  Given contributions from potentially multiple institutions, intersectoral action can be complex.  Evaluation activities should be based on a theory of change so that relevant processes and outcomes can  be identified and assessed. |
| 14a, 14b | Modification of collaborative action over time based on evaluation. | Evidence from evaluations is used to improve existing interventions or introduce new interventions. | Evidence-informed modifications of existing interventions can improve specific shortcomings of the initial intervention design. Evidence from evaluation may also indicate a need for new interventions that target unaddressed aspects of health inequities. |

4

Glossary Intervention

An intervention is an action or course of actions planned with the goal of changing a process or outcome in order to promote health or health equity.

Upstream approach

The upstream approach to health equity is an approach that focuses on macro-level interventions that examine the root cause of inequity and build interventions rooted in policy and government changes (Bharmal et al., 2015). An upstream approach to health addresses the social determinants of health, such as education and income.

Midstream approach

Midstream approaches are those that focus on intermediary determinants, such as housing, employment and food security (NCCD, 2014). These interventions reduce group vulnerabilities and focus on changing the cause of the problem. Midstream interventions happen at the micro-level, such as in communities and neighbourhoods (NCCD, 2014).

Downstream approach

A downstream approach is focused on micro-level strategies for health and has a greater focus on access to healthcare resources compared to the root cause of the health disparity (NCCD, 2014). These interventions provide services to individuals who are already facing a health disparity.

Social determinants of health

The social determinants of health are non-medical factors that affect individual and population health. The WHO defines the social determinants of health as “the conditions in which people are born, grow, live, work and age,” which can affect health behaviours and outcomes (Braveman & Gottlieb, 2014).

Structural determinants of health

The structural determinants of health are the factors that “shape the quality of the social determinants of health” such as the political, legal and economic policies that effect working conditions, housing, education etc.

5
